# Complicated grief and its relationship with anxiety, depression, and suicidal ideation in older adults in the context of the COVID-19 pandemic in Peru: a cross-sectional analysis

**DOI:** 10.1186/s12888-023-05412-5

**Published:** 2023-12-05

**Authors:** Stefan Escobar-Agreda, Zoila Romero Albino, Pavel J. Contreras, María Sofía Cuba-Fuentes

**Affiliations:** 1 Unidad de Telesalud, Universidad Nacional Mayor de San Marcos, Lima, Peru; 2 Elderly Protection Direction, EsSalud, Lima Peru; 3 Padomi Children, Flexible Offer Management, Essalud, Lima Peru; 4 Centro de Investigación en Atención Primaria de Salud, Universidad Peruana Cayetano Heredia, 775 Jose Gonzales Street, Apt. 604, Miraflores, Lima, Perú; 5 Juan Jose Rodriguez Lazo Polyclinic, EsSalud, Lima Perú

**Keywords:** Complicated grief, Older adults, Anxiety, Depression, suicidal ideation, COVID-19

## Abstract

**Background:**

Complicated grief (CG) resulting from poor adaptation to the death of a close person may have been related with the presence of other mental health problems in older adults in Peru during the COVID-19 pandemic. Our study aimed to assess the association between CG and anxiety, depression, and suicidal ideation in older adults in Peru in the context of the COVID-19 pandemic.

**Methods:**

We conducted a cross-sectional analysis using data from the “Socioemotional evaluation form” applied in 2020 to mental health problems in older adults attending the Peruvian Social Security (EsSalud). For our study, we included older adults who reported the death of a close person during the last six months when this assessment was performed. CG, depression, anxiety, and suicidal ideation were initially evaluated using validated questionnaires. The association between CG and the presence of mental health problems was calculated through multivariate analysis, where prevalence ratios (PR) were estimated with 95% confidence intervals (CI).

**Results:**

Of the 249 older adults included, 175 (70.3%) were female with a median age of 71 years (interquartile range: 9), and 35 (14.1%) reported the presence of CG. It was found that CG in this population was associated with the presence of anxiety (PR: 1.35, 95% CI: 0.98 to 1.85), depression (PR: 1.44, 95% CI: 1.06 to 1.95), and suicidal ideation (PR: 2.84, 95% CI: 1.06 to 7.59).

**Conclusions:**

CG is related to the presence of mental health problems in older adults in Peru. It is essential to implement measures that facilitate the prevention and proper management of this condition in this population, especially in the context of high population mortality such as the COVID-19 pandemic.

## Background

The COVID-19 pandemic significantly impacted the physical and mental health of the older adult population worldwide [1]. These have been particularly affected by the pandemic, as they are not only more vulnerable to the disease but have also had their social, work, and leisure activities affected by mandatory social isolation [2]. As a result, mental health problems have emerged in this population, such as anxiety, depression, and suicidal ideation, as demonstrated in previous studies [3, 4].

In addition, the increased death of relatives or close people to older adults during the pandemic has led to an increase in conditions such as complicated grief (CG) [5], which is described as a maladaptation to the loss of a close person that generates significant interference in the affected person’s life. While various studies have explored the factors related to the presence of complicated grief [6, 7], there is currently limited evidence on the consequences of this condition, especially in the presence of mental health problems such as anxiety, depression, or suicidal ideation in the older adult population during COVID-19 pandemic.

Peru, one of the countries most affected by the pandemic, has reported more than 200,000 COVID-19 deaths, of which 70% correspond to people over 60 [8]. In this context in Peru, in 2020, the Elderly and Social Benefits Management of the Social Security of EsSalud implemented an accompaniment strategy for older adults that included evaluating socioemotional aspects, including mental health conditions in insured older adults. Based on this information, the present study aims to evaluate the relationship between CG and the presence of mental health problems such as anxiety, depression, and suicidal ideation in the older adult population during the COVID-19 pandemic.

## Methods

### Study design and setting

We conducted an observational cross-sectional study performing a secondary database analysis using national data from the “Socio-Emotional Assessment Form” applied to older adults from September to November 2020 by the Management of the Elderly Person and Social Benefits (GPAMYPS) of the Peruvian Social Security (EsSalud). In Peru, EsSalud serves as a key healthcare provider and insurance entity for employees and their dependents who are part of the formal labor market, covering approximately 35% of the country’s total population [9]. Meanwhile, the Ministry of Health (MoH) of Peru oversees public healthcare services for the remaining population, offering coverage through the Integral Health Insurance program, which is specifically designed to assist uninsured citizens, particularly those who are economically disadvantaged or otherwise vulnerable.

### Population and sample

In our study, we included information on older adults (≥ 60 years) assessed through the Socio-Emotional Assessment Form that have reported the death of a family member or a close person during the last six months since the realization of this assessment.

### Variables

#### Dependent variables: mental health problems

We included information from the Socio-Emotional Assessment Form for older adults, including mental health problems. The evaluation of depression was performed using the PHQ-9 scale validated in Peru [10, 11]. We categorized this variable according to the presence of depression (5 points or more on the PHQ-9 scale) and according to its severity level as “mild” (5 to 9 points), “moderate” (10 to 14 points), “severe” (15 to 19 points), or “very severe” (20 to 27 points). This tool has shown good reliability among Peruvian population (Cronbach’s alpha = 0.89; McDonald’s omega = 0.86), and had a sensitivity of 76.0% and a specificity of 72.1% for major depression when the score is ≥ 7 points [12].

The evaluation of anxiety was assessed through the GAD-7 scale validated in Spanish [13]. We categorized this variable according to the presence of anxiety (5 points or more on the GAD-7 scale) and according to its severity level as “mild” (5 to 9 points), “moderate” (10 to 14 points), or “severe” (15 to 21 points). This tool also has shown good reliability among Peruvian population (Cronbach’s alpha = 0.85; McDonald’s omega = 0.81), and had a sensitivity of 53.6% and a specificity of 78.8% for anxiety when the score is ≥ 8 points [12].

**Table 1:** Sociodemographic and mental health characteristics of the older adults evaluated. Peru, 2020

	No.	%
Total	249	100.0%
Age (Median, IQR)	71.0	(9.0)
Categorized age		
60–69 years	76	30.8%
70–79 years	133	53.8%
≥80 years	38	15.4%
No data	2	< 0.1%
Sex		
Female	175	70.3%
Male	74	29.7%
Cohabitation		
Live alone	6	2.4%
Live with one person	53	21.4%
Live with two or more people	189	76.2%
No data	1	< 0.1%
Region where is attended		
Lima	187	77.6%
Other regions	54	22.4%
No data	8	< 0.1%
Complicated grief (CG)		
With CG	35	14.1%
Without CG	214	85.9%
Level of Depression		
No depression	161	64.7%
Mild depression	55	22.1%
Moderate depression	13	5.2%
Moderately severe depression	5	2.0%
Severe depression	15	6.0%
Level of Anxiety		
No anxiety	163	65.5%
Mild anxiety	54	21.7%
Moderate anxiety	20	8.0%
Severe anxiety	12	4.8%
Suicidal ideation		
With suicidal ideation	16	6.8%
Without suicidal ideation	221	93.2%
No data	2	< 0.1%

The evaluation of suicidal ideation was performed through questions related to the presence of suicidal thoughts or attitudes in the last two weeks based on the recommendation of Park et al. [14]. Those who affirmed presenting any of these conditions were categorized as “With suicidal ideation”, and the rest as “Not suicidal ideation”. This tool has shown good validity and reliability scores (content validity index = 0.89, content validity ratio = 0.85, Cronbach’s alpha = 0.76) among older adults [15].

#### Independent variable: complicated grief

The complicated grief was evaluated using the Brief Complicated Grief Scale, which includes attitudes or states of grief following the loss of a close person or family member in the last two weeks. This variable was categorized as “with CG” and “without CG” Each question is scored from 0 to 2 points, where 0 = not at all, 1 = somewhat and 2 = very much. A score of 4 or more indicates risk of complicated grief. This tool has shown good a discriminant validity and reliability scores (Average Variance Extracted = 0.39, Cronbach’s alpha = 0.75) [16].

**Table 2:** Characteristics of older adults according to the presence of anxiety, depression, and suicidal ideation. Peru, 2020

	Anxiety			Depression			Suicidal ideation		
	No Presence	Presence	*p-value*	No Presence	Presence	*p-value*	Without SI	With SI	*p-value*
	*n (%)*	*n (%)*		*n (%)*	*n (%)*		*n (%)*	*n (%)*	
Total	163	86		161	88		221	16	
Age (Median, IQR)	72 (8.0)	71 (9.0)	0.09^1^	71.5 (8.0)	71 (9.0)	0.54^1^	72 (8.0)	71 (9.0)	0.90^1^
Categorized age									
60–69 years	44 (27.2)	32 (37.6)	0.19^2^	45 (28.1)	31 (35.6)	0.40^2^	66 (29.9)	5 (33.3)	0.95^2^
70–79 years	90 (55.6)	43 (50.6)		91 (56.9)	42 (48.3)		121 (54.8)	8 (53.3)	
≥80 years	28 (17.3)	10 (11.8)		24 (15.0)	14 (16.1)		34 (15.4)	2 (13.3)	
Sex									
Female	106 (65.0)	69 (80.2)	0.01^2^	109 (67.7)	66 (75.0)	0.23^2^	154 (69.7)	13 (81.2)	0.32^2^
Male	57 (35.0)	17 (19.8)		52 (32.3)	22 (25.0)		67 (30.3)	3 (18.8)	
Cohabitation									
Live alone	3 (1.8)	3 (3.5)	0.57^3^	3 (1.9)	3 (3.4)	0.64^3^	6 (2.7)	0 (0.0)	0.42^3^
Live with one person	33 (20.2)	20 (23.5%)		33 (20.5)	20 (23.0)		45 (20.4)	5 (33.3)	
Live with two or more people	127 (77.9)	62 (72.9)		125 (77.6)	64 (73.6)		170 (76.9)	10 (66.7)	
Region where patient is attended									
Lima	34 (21.4)	20 (24.4)	0.60^2^	33 (21.0)	21 (25.0)	0.49^2^	46 (21.4)	4 (28.6)	0.55^2^
Other regions	125 (78.6)	62 (75.6)		124 (79.0)	63 (75.0)		169 (78.6)	10 (71.4)	
Complicated grief (CG)									
With CG	13 (8.0)	22 (25.6)	< 0.001^2^	11 (6.8)	24 (27.3)	< 0.001^2^	24 (10.9)	7 (43.8)	< 0.001^2^
Without CG	150 (92.0)	64 (74.4)		150 (93.2)	64 (72.7)		197 (89.1)	9 (56.2)	

IQR: interquartile range

^1^Mann Whitney U test

^2^Fisher’s exact test

^3^Chi-square test

#### Covariates

As intervening variables, we considered the sociodemographic characteristics of the population studied, including sex categorized as “female” and “male,“ age evaluated in years numerically and through categories: “60–69,“ “70–79,“ “80–89,“ and “90–99,“ and the region where the older adult was evaluated categorized as “Lima” and “other regions.“ Additionally, we included information on older adult’s cohabitation, categorized as “lives alone,“ “lives with one person,“ and “lives with two or more people.“

### Data collection procedures

The Socio-Emotional Assessment for older adults was an initiative deployed by the GPAMYPS to assess prevalent mental health problems among older adults from the Peruvian Social Security (EsSalud) during the COVID-19 pandemic. The primary objective was to gather screening data on the mental health of older adults, with a specific focus on those who had exhibited negative mental health symptoms (e.g., sadness, anxiety, stress, fear) during remote monitoring sessions amidst the pandemic in line with the GPAMYPS’s Institutional Operational Plan.

**Fig. 1:**
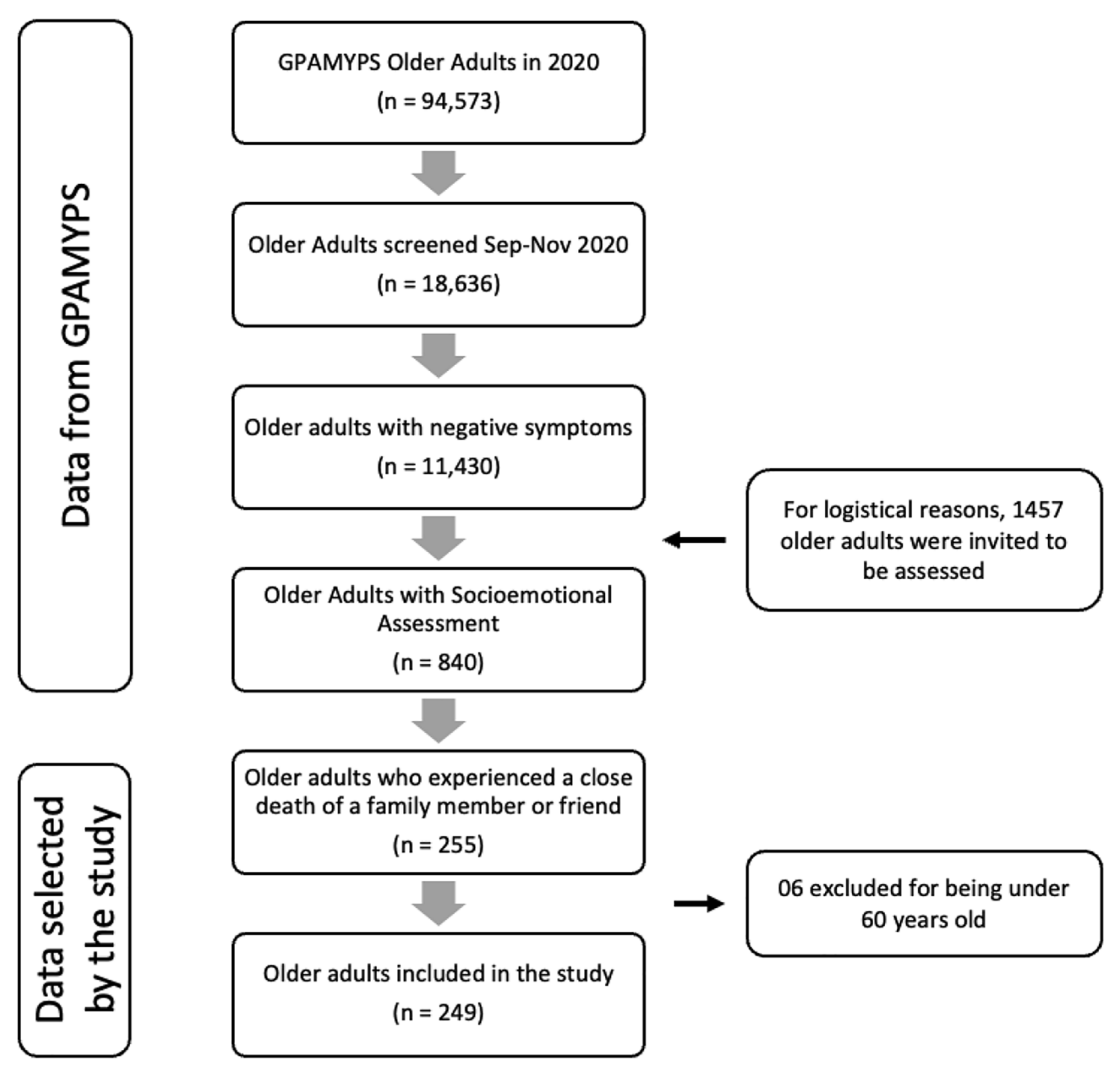
Flowchart of older adults selected to the study

Given logistical constraints, out of the 11,430 older adults with negative mental health symptoms monitored between September and November 2020, EsSalud evaluated a sample of 1,457 individuals (Fig. 1). Of these, 840 agreed to complete the “Socioemotional Evaluation Form”. For our study, we initially considered 255 participants who had experienced the death of a close family member or friend within the two weeks leading up to this evaluation. We then excluded six individuals below 60, resulting in a final sample of 249 older adults for analysis.

The Socio-Emotional Assessment’s data was collected via telephone by trained EsSalud personnel, who collected the information digitally using previously validated questionnaires. A de-identified version of this database was subsequently provided to our research team.

### Statistical analysis

We performed a descriptive analysis of the older adults evaluated. We used the median and interquartile range to describe the numerical variable age, given its skewed distribution, and we used the frequencies and proportions to describe the remaining categorical variables. Additionally, we performed a bivariate analysis where we explored the relationship between sociodemographic characteristics, the presence of CG and anxiety, depression, and suicidal ideation in older adults evaluated through Fisher’s exact test or Chi-square test according to each case.

To evaluate the association between CG and the presence of anxiety, depression, and suicidal ideation, multivariate analyses were performed using Poisson regressions with robust variance, obtaining crude and adjusted prevalence ratios (PR) with 95% confidence intervals (95% CI). Confounding variables for each model included the sociodemographic variables (gender, age, place of origin, and cohabitation) and the coexistence of mental health problems according to each case based on the theoretical framework. All statistical analyses were performed using Stata v17.0 software [17].

## Results

Table 1 shows the sociodemographic and mental health characteristics of the population studied. Among them, 175 (70.3%) were female with a median age of 71 years (interquartile range: 9). Additionally, 243 (97.6%) reported living with at least one person, and 187 (77.6%) were part of the health network in Lima. Regarding mental health conditions, 35 (14.1%) of the elderly adults manifested the presence of complicated grief, 88 (35.3%) the presence of depression, 86 (34.5%) the presence of anxiety, and 16 (6.8%) a suicidal ideation.

**Table 3:** Association of complicated grief and the presence of anxiety, depression, and suicidal ideation in older adults. Peru, 2020

		Complicated grief (CG)	
		Without CG	With CG PR (95% CI)
Presence of Anxiety			
	Crude	Ref	2.10 (1.51 to 2.92)
	Adjusted^1^	Ref	1.35 (0.98 to 1.85)
Presence of Depression			
	Crude	Ref	2.29 (1.69 to 3.11)
	Adjusted ^2^	Ref	1.44 (1.06 to 1.95)
Suicidal ideation			
	Crude	Ref	5.17 (2.07 to 12.90)
	Adjusted ^3^	Ref	2.84 (1.06 to 7.59)

PR: Prevalence ratios, 95% CI: 95% confidence interval

Baseline confounders: sex, categorized age, region of origin, cohabitation

Presence of anxiety (≥ 5 points in GAD-7), Presence of depression (≥ 5 points in PHQ-9), Suicidal ideation (Affirm at least one question of the Park et al. questionnaire)

^1^Adjusted for baseline confounders and presence of depression

^2^Adjusted for baseline confounders and presence of anxiety

^3^Adjusted for baseline confounders and presence of anxiety and depression

The bivariate level comparisons between the sociodemographic characteristics and CG and the presence of anxiety, depression, or suicidal ideation in the elderly adults are shown in Table 2. The study found a relationship between age, sex and the presence of risk of anxiety and a relationship between the presence of CG and the presence of anxiety, depression, and suicidal ideation in this population.

Table 3 shows the results of the evaluated associations between the presence of CG and the presence of anxiety, depression, and suicidal ideation in elderly adults using multivariate analyses. The study found among this population a significant association between the presence of CG and depression (PR: 1.44, 95% CI: 1.06 to 1.95), and suicidal ideation (PR: 2.84, 95% CI: 1.06 to 7.59) and a potential relation with CG and the presence of anxiety (PR: 1.35, 95% CI: 0.98 to 1.85).

## Discussion

The current study found a relationship between complicated grief (CG) and depression, anxiety, and suicidal ideation in older adults. Our results are consistent with previous literature findings. For instance, a nationwide cohort study conducted by Guldin et al. in Denmark (2016) demonstrated that individuals who experienced the loss of a close relative had a greater risk of suicide, deliberate self-harm, and psychiatric illness than the general population [18]. CG was identified as a significant risk factor for depression in the population studied. Additionally, Szanto et al. (2020) in the United States found that CG severity was a significant predictor of suicidal ideation in elderly bereaved individuals [19]. Finally, Dominguez-Lopez et al. (2021) reported that grief in Mexican older adults seeking psychological support during the COVID-19 pandemic was significantly associated with higher anxiety symptoms than in younger adults [20].

Several explanations have been proposed in the literature to account for the associations observed in our study. For example, disruptions in the neural circuitry involved in emotion regulation and reward processing may contribute to the high rates of comorbidity between CG and depression, as noted by LeBlanc et al. [21]. Complicated grief may lead to negative self-evaluations and feelings of guilt, dysregulation of the hypothalamic-pituitary-adrenal axis that regulates the body’s stress response, and difficulty regulating emotions, all of which may contribute to the development of anxiety [22–24]. The emotional pain, sense of hopelessness and despair associated with CG, as well as the loss of meaning and purpose, social isolation, and withdrawal may also increase the risk of suicidal ideation [19].

While the associations between CG and mental health problems such as depression, anxiety, and suicidal ideation have been observed across all age groups, they may be particularly pronounced in older adults. Older adults may be more affected by the cumulative effect of multiple losses and have fewer resources and outlets for coping. This vulnerability may be exacerbated by the COVID-19 pandemic, which has caused many deaths among elders and increased the frequency of CG in this population. Indeed, in our study, 14.1% of the older adults evaluated showed signs of CG, which is higher than in previous studies [25]. This may explain the increasing prevalence of depression, anxiety, and suicidal ideation among older adults during the COVID-19 pandemic, as reported in previous studies [26].

Our results underscore the need for clinicians and caregivers to be knowledgeable about CG and provide appropriate support and treatment to grieving older adults. Early detection and intervention for CG should be a priority to prevent or reduce the severity of these mental health conditions [27]. Furthermore, therapies designed to manage CG in older adults, such as cognitive-behavioral therapy, have also been shown to be effective in reducing symptoms of depression [28], and mindfulness interventions have been found to reduce symptoms of depression and anxiety [29].

Some limitations to our study should be taken into consideration when interpreting our findings. First, our sample only included older adults who were part of the Social Security System in Peru, which may limit the generalizability of our findings to broader populations. Additionally, the lack of information about specific covariates, such as socioeconomic status, caregiver presence, chronic conditions or disabilities, and past mental health issues, may impact the accuracy of our results regarding the relationship between CG and mental health problems in older adults. Moreover, information about the sampling criteria used on the original assessment of older adults made by EsSalud was unavailable, which could influence the representativeness of our results. Finally, our study also relied on self-reported measures to assess mental health conditions among older adults, which may be subject to biases inherent in each instrument used. Despite these limitations, our work sheds light on the relationship between CG and mental health problems among older adults in Latin American low-middle-income countries such as Peru. The associations observed align with those from other studies, reinforcing their global relevance, especially in high-mortality contexts like the COVID-19 pandemic.

## Conclusions

Our findings highlight the importance of screening for CG in the elderly population, especially those with these mental health conditions. Further research is needed to explore the factors that mediate these associations, the magnitude of CG conditions, and the appearance of these conditions by using longitudinal study designs. Our results also recall the importance designing and deploying effective interventions that could prevent the development of complicated grief and problems such as depression, anxiety, or suicidal ideation in older adults, especially in high mortality contexts such as disasters or pandemics.

## Data Availability

The datasets generated and analyzed during the current study are not publicly available as they are part of confidential information of patients attended in EsSalud but could be anonymized and shared from the corresponding author at a reasonable request.

## List of Abbreviations

CG: Complicated Grief
GAD-7: Generalized Anxiety Disorder 7-item scale
PHQ-9: Patient Health Questionnaire-9
EsSalud: Peruvian Social Security
GPAMYPS: Management of the Elderly Person and Social Benefits
